# The role of diabetes mellitus on the formation of severe odontogenic abscesses—a retrospective study

**DOI:** 10.1007/s00784-021-03926-4

**Published:** 2021-05-13

**Authors:** Roman Kia Rahimi-Nedjat, Keyvan Sagheb, Kawe Sagheb, Maike Hormes, Christian Walter, Bilal Al-Nawas

**Affiliations:** 1 Department of Oral and Maxillofacial Surgery – Facial plastic Surgery, Medical Center of the Johannes Gutenberg-University of Mainz, Augustusplatz 2, 55131 Mainz, Germany; 2 Department of Prosthodontics, Medical Center of the Johannes Gutenberg-University of Mainz, Mainz, Germany

**Keywords:** Odontogenic abscesses, Diabetes mellitus, Blood sugar count, Abscess treatment

## Abstract

**Objectives:**

To analyze the correlation of diabetes mellitus and hyperglycemia with severe odontogenic abscesses.

**Materials and methods:**

Records of all patients in the Department of Oral and Maxillofacial Surgery of the Medical Center of the Johannes Gutenberg-University who underwent inpatient treatment for severe odontogenic abscesses between 2010 and 2016 were evaluated retrospectively regarding diabetes anamnesis, maximum and fasting blood sugar count, and duration until discharge. In order to compare the numbers to a general maxillofacial group, all patients who received inpatient treatment in 2013 for any diagnosis other than an abscess of the head and neck region were analyzed as well, and the numbers were correlated.

**Results:**

In total, 977 abscess patients were found in the analyzed period. 7.0% of the patients had a known diagnosis of diabetes mellitus type II and 0.6% of type I. Correlation with the general group showed that abscesses were significantly more likely in diabetics as well as patients with abnormal maximum and fasting blood sugar counts. These patients also needed significantly longer inpatient treatment.

**Conclusions:**

Diabetics and patients with abnormal glucose tolerance show significantly higher numbers of severe odontogenic abscesses and might therefore benefit from earlier escalation of antibiotic medication.

**Clinical relevance:**

Severe odontogenic abscesses are one of the most frequent diagnoses in maxillofacial practice. Adjusting the therapeutic approach for diabetics or patients with abnormal blood sugar counts might help to prevent the development of abscesses.

## Introduction

The state in which a person suffers an absolute or relative reduction of insulin is known as diabetes mellitus. In addition to typical symptoms such as frequent urination and thirst and unspecific symptoms such as fatigue or recurrent infections, these patients show abnormal blood sugar counts with elevated fasting glucose tolerance above 126mg/dl. With more than 400 million affected patients, diabetes mellitus is one of the largest medical epidemics of the present time. Due to unhealthy nutrition, lack of exercise, and consequent obesity, the diagnosis can increasingly be found even among younger individuals, but in general, diabetes mellitus is most frequent in patients over 60 years of age [1–3].

Besides sociomedical issues, comorbidities and illnesses are a large burden for both the patients and the national health care systems. Early diabetes-associated pathologies of the cardiovascular and neural system can be found years prior to its manifestation and usually have already developed irreversible damage at the time of diagnosis. Lowered immune defense and recurrent infections usually are part of late onset diabetes [4–6].

Abscesses are one of the most frequent diagnoses in the maxillofacial practice, with most originating from odontogenic infections. While the vast majority of the cases can be treated sufficiently by a dentist, for example through local incision or calculated antibiotic treatment, some infections tend to progress and form a severe abscess which then requires inpatient treatment with intravenous antibiotic treatment and extended surgical intervention, depending on the abscess’ extent, location, and the patients’ anamnesis [7, 8].

The treatment of abscesses is usually not a great challenge nowadays. Yet, some patients show more complicated courses of disease with longer inpatient stays and faster progression of the infection onto different head and neck regions at the time of admission. As diabetes mellitus tends to compromise immune response and therefore makes patients prone to infections, one might expect it to have an influence on abscess formation. Therefore, it was our objective to investigate the relationship between diabetes and severe odontogenic abscesses and whether diabetics show more complicated disease progressions.

## Material and methods

A retrospective case-control study was conducted to test the following hypotheses:

I. Patients with a known diagnosis of diabetes mellitus or abnormal glucose tolerance have a higher risk to form a severe abscess than non-diabetics.
II. Patients with diabetes-mellitus or abnormal glucose tolerance need longer inpatient treatment.

All patients who underwent inpatient treatment due to a severe odontogenic abscess in the years 2010 to 2016 in the Department of Oral and Maxillofacial Surgery of the Medical Center of the Johannes Gutenberg-University of Mainz were retrospectively included in this study. A severe abscess was defined as any infection exceeding its local borders with wide involvement of soft tissue compartments.

The study was conducted according to the declaration of Helsinki and the ethical guidelines of the federal hospital law of Rhineland-Palatinate.

Electronic health records were evaluated for the following details:

- Demographic data such as gender and age
- Location of the abscess
- Diabetes anamnesis (type I or type II)
- Type of diabetes therapy (medicinal or non-medicinal)
- Anamnesis of typical diabetes related illnesses
- Abnormal glucose tolerance was captured by two separate values:
  - Maximum blood sugar count (MBSC) during the inpatient stay (a blood sugar count over 200mg/dl at any time was defined as abnormal [9])
  - Fasting blood sugar count (FBSC) (fasting blood sugar count was measured only in the morning before breakfast and was defined as abnormal above 126mg/dl [9])
- Duration of inpatient treatment

To be able to compare the data of the abscess patients with those of a general maxillofacial patient group, all cases who underwent inpatient treatment because of any other diagnose during the year 2013 were analyzed as well for criteria mentioned above. All patients who had an incomplete electronic health record for any of the information except the FBSC were excluded from our analysis.

All data were imported into SPSS 22.0 (IBM SPSS Inc., Chicago, IL, USA). In addition to a descriptive analysis, chi-squared tests were used to determine the association between nominal variables. For the correlation of mean values of parametrically distributed continuous variables, the *t*-test was used, and the and Kruskal-Wallis-test was used for non-parametrically distributed continuous variables. Odds ratios were calculated with a mathematical formula in excel. A *p*-value of <0.05 was considered as statistically significant.

## Results

### Patients

**Fig. 1:**
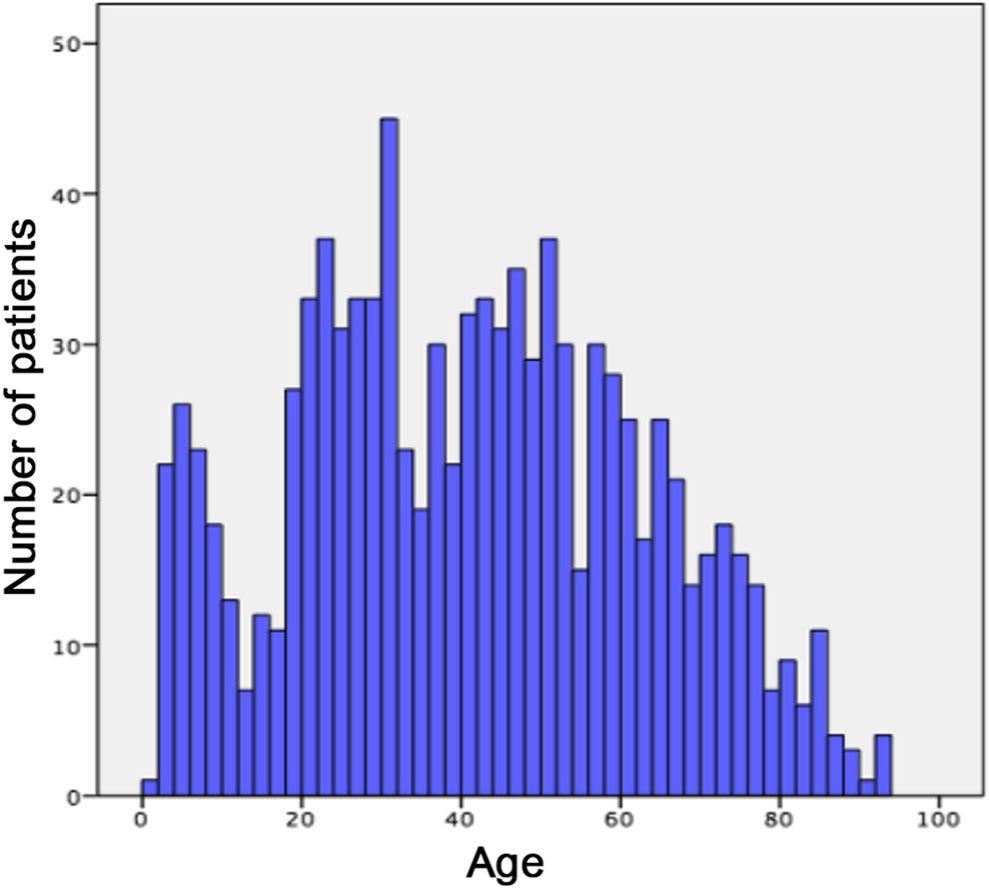
Age distribution of the abscess patients in absolute numbers

In total, 977 patients with severe odontogenic abscesses were found in the observed period, with a mean age of 41 years (±21.5years). A total of 538 patients were male (55.1%) and 439 female (44.9%). With a mean age of 39.2 years, the female patients were slightly younger than the males, who were 43.2 years old on average (*p* = 0.004). Most patients who presented with a severe odontogenic abscess were between 20 and 29 years of age (17.1%) (Fig. 1).

### Diabetes anamnesis and blood sugar counts

In the abscess group, diabetes mellitus was known among 7.3% of the 977 patients (*n* = 71). From these, 6 patients had type I diabetes.

**Fig. 2:**
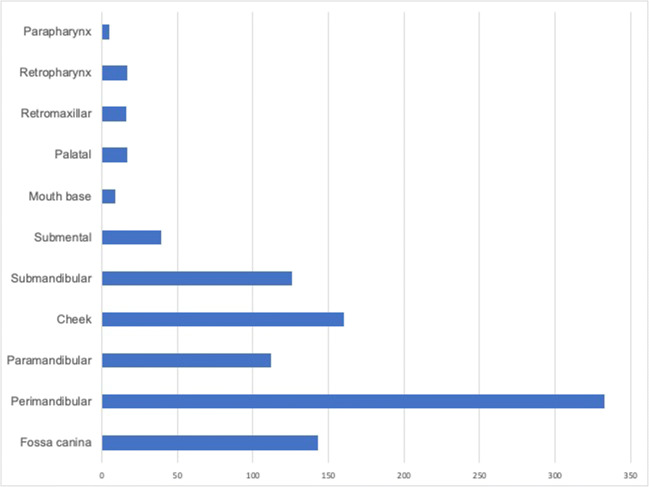
Involved compartments in absolute numbers

Among all 977 patients, an abnormal MBSC was found in 5.7% (*n* = 56), of whom only 32 (57.1%) were known to have diabetes. This means that 42.9% of these patients had an impaired glucose tolerance but had not been diagnosed with diabetes. Out of all abscess patients, 39 showed an increased FBSC (4.0 %) and of these, 22 (56.1%) already had a diabetes diagnosis (Table 1, Fig. 2).

**Table 1:** Overview of age, gender ratio, and duration of hospital stay in the abscess group. Number of patients with abnormal maximum blood sugar counts (MBSC) and fasting blood sugar counts (FBSC). Distribution of the abscesses to the different compartments

	Non-diabetics		Diabetics		All	
Age (SD, min–max)	39.4	(±21.1, 1–93)	61.1	(±16.8, 20–92)	41	(±21.5)
Male	501	(55.3%)	37	(52.1%)	538	(55.1%)
Female	405	(44.7%)	34	(47.9%)	439	(44.9%)
*N*	906	(92.7%)	71	(7.3%)	977	(100%)
Mean hospital stay (SD)	6 days	(±3 days)	6.4 days	(±3.3 days)	6 days	(±3 days)
Type I diabetes			6	(8.5%)	6	(8.5%)
Type II diabetes			65	(91.5%)	65	(91.5%)
Medicinal treatment			59	83.1%)	59	83.1%)
Dietary treatment			12	(16.9%)	12	(16.9%)
Abnormal MBSC	24	(42.9%)	32	(57.4%)	56	(5.7%)
Abnormal FBSC	17	(47.2%)	22	(56.4%)	39	(3.9%)
Perimandibular	316	(34.9%)	17	(23.9%)	333	(34.0%)
Cheek	149	(16.5%)	11	(15.5%)	160	(16.4%)
Submental	28	(3.1%)	11	(15.5%)	39	(4.0%)
Paramandibular	102	(11.3%)	10	(14.1%)	112	(11.5%)
Fossa canina	134	(14.8%)	10	(14.1%)	144	(14.7%)
Submandibular	135	(14.9%)	10	(14.1%)	145	(14.8%)
Mouth base	8	(0.9%)	1	(1.4%)	9	(0.9%)
Palatal	11	(1.2%)	1	(1.4%)	12	(1.2%)
Retromaxillar	10	(1.2%)	1	(1.4%)	11	(1.1%)
Parapharynx	1	(0.1%)			1	(0.1%)
Retropharynx	12	(1.3%)			12	(1.2%)

The mean FBSC for all abscess patients was 114.0 mg/dl and the mean MBSC 112.9 mg/dl. The diabetes patients in this group showed higher blood sugar counts (FBSC 154.5 mg/dl, MBSC 234.1 mg/dl, Table 2)

**Table 2:** Mean FBSC and MBSC counts for patients from the abscess group (a) and patients from the general group (g). Values in mg/dl and standard deviation in brackets

	FBSC (mean ±SD)	FBSC (min–max)	MBSC (mean ±SD)	MBSC (min–max)
All (a)	114.0 ±73.2	43–478	112.9 ±71.4	39–784
Diabetics (a)	154.5 ±76.7	80–394	243.1 ±145.2	64–784
Non-diabetics (a)	101 ±64.1	43–478	101.9 ±47.2	39–655
all (g)	105.7 ±44.1	30–350	109.0 ±73.5	34–600
Diabetics (g)	137.0 ±62.8	30–350	231.0 ±114.5	76–600
Non-diabetics (g)	90.0 ±33.8	50–267	107.1 ±63.2	34–600

Of the 71 diabetes patients, 59 received medicinal treatment while 12 managed their diabetes with diet. The most frequent and almost only diabetes-related disease in the anamnesis was nephropathy and could be found in 14 cases. Three more patients had a history of retinopathy and one of neuropathy.

### Severe odontogenic abscesses

With 34.1%, the perimandibular compartments were the most frequent localization of severe odontogenic abscesses (Table 1), followed by the cheek (16.4%), and fossa canina (14.6%). The mean inpatient stay was an average of 6 days (±3 days) for all patients. While there was no significant difference for the hospital stay between diabetics and non-diabetics (*p* = 0.387, median inpatient stay of 6.4 days), we found a significantly longer hospitalization for patients with abnormal MBSC (*p* = 0.046, median inpatient stay of 7.5 days) and FBSC (*p* 0= 0.008, median inpatient stay of 9.2 days).

### The general group

The investigation of the general group from 2013 involved 2258 patients (Table 3). These patients had a mean age of 48.0 years (±23.7years). The proportion of diabetics was 5.3% (*n*=121). Abnormal MBSC was found in 10.7% (*n*=242) and impaired FBSC in 8.2% (185). Here again these numbers are higher than the number of diabetics since not every patient with an impaired glucose tolerance had been diagnosed with diabetes. For this group, the mean FBSC was 105.7 mg/dl and MBSC was 109.0 mg/dl. These numbers were slightly below those found in the abscess patients (Table 2)

**Table 3:** Overview of age, gender ratio, and duration of hospital stay in the general group (non-abscess patients). Number of patients with abnormal maximum blood sugar counts (MBSC) and fasting blood sugar counts (FBSC)

	Non-diabetics			Diabetics			All	
Age (SD)	49.9	(±22.9)		69.2	(±13.2)		50.1	(±22.9)
Male (%)	1260	(59%)		81	(66.9%)		1341	(59.4%)
Female (%)	877	(41%)		40	(32.5%)		917	(40.6%)
*N* (%)	2137	(94.6%)		121	(5.3%)		2258	(100%)
Mean hospital stay	3 days	(±5.4 days)		7 days	(±6.9 days)		3 days	(±5.5 days)
Type I diabetes				4	(3.3%)		4	(3.3%)
Type II diabetes				117	(96.7%)		117	(96.7%)
Dietary treatment				28	(23.1%)		28	(23.1%)
Medicinal treatment				93	(76.9%)		93	(76.9%)
Abnormal MBSC (%)	162	(66.9%)		80	(33.1%)		242	(10.7%)
Abnormal FBSC (%)	122	(65.9%)		63	(34.1%)		185	(8.2%)

### Comparison of the abscess and general patients

Comparison of the mean ages shows that patients with abscesses were an average 9 years younger than the general group, and diabetics were significantly older than all other patients (*p* < 0.001).

The portion of diabetics among patients under 60 years of age in the abscess group was twice as high as those under 60 in the general group (4.6%, *n* = 36 versus 2.1%, *n* = 29). This difference was highly significant (*p* < 0.001). In patients older than 60, these numbers were opposite as the portion of diabetics was higher in the general group with 19.1% compared to 17.7% in the abscess group. However, this difference was not significant.

Finally, adding all patients from both groups into one group and then dividing them into diabetics and non-diabetics allows an examination of the portion of abscesses in both groups. This calculation shows a significant difference, with a higher number of abscesses in diabetics (*p* = 0.025). An even higher significance was found for those with abnormal MBSC and FBSC (*p* < 0.001).

This relationship could also be observed in the odds ratio. The occurrence of a severe abscess in diabetics was 1.28 times more likely than in non-diabetics. This number was even higher for diabetics with impaired FBSC (2.51) and for those with abnormal MBSC (2.7).

## Discussion

The aim of this study was to investigate the influence of diabetes and abnormal glucose counts on severe odontogenic abscesses. For this purpose, two different patient groups were analyzed. The first group consisted of all patients who received inpatient treatment due to a severe odontogenic abscess in the years 2010 to 2016; the second was a general group made of all patients who received inpatient treatment during the year 2013. The general group involved all diagnoses other than odontogenic abscesses or any other abscesses in the head and neck region.

Our study revealed 977 abscess patients in the observed period, which were compared to 2258 patients from the general group. With 7.3%, the number of diabetics among our abscess patients was low compared to other studies, which have found diabetes in about 20–41% of cases with deep neck infections [10–12].

One reason for the difference might be the age of the patients. In our study, we focused only on odontogenic abscesses. Generally, these patients are younger than those with deep neck infections. The fact that the prevalence of diabetes increases with age may explain our lower percentage of diabetic patients. Hence, when focusing only on patients who were older than 60, the number of diabetics in our study became considerably higher and thus similar to reports in the literature. Unfortunately, as the mentioned studies focus on all neck abscesses and not only odontogenic infections, it is not possible for us to fully compare the diabetes ratio with our findings.

It is a well-established fact that diabetics have an impaired immune response compared to non-diabetics. Among many different factors, this is due to macro- and micro-angiopathies that lead to delayed and obstructed granulocyte invasion. Since cell migration is a fundamental basis in the wound healing cascade, it is not surprising that in our study a significant difference could be observed for the duration of inpatient treatment among patients with abnormal MBSC and FBSC. Yet, the difference was not significant for diabetics versus non-diabetics. Similar observations, however, can also be found in the literature. As well, in a previous study of own, we were able to show that while diabetics have a worsened immune response and therefore are prone to infections, they can have regular wound healing and a normal treatment process with similar inpatient stays if they are under good medicinal or dietary treatment and therefore maintaining normal blood sugar counts [13–16].

The relevance of the blood sugar count can also be seen when joining the two groups and then dividing them again according to diabetic status. There is a significant difference in the incidence of abscesses, with higher a number among diabetics over non-diabetics. Yet, the difference is even more significant for patients who showed abnormal blood sugar counts compared to those with normal values. These findings are underlined in the calculation of the odds ratio which shows that abscesses are more likely to occur in diabetics and particularly among those with abnormal blood sugar counts. This is in concordance with our previous findings as well as the study by Wong et al. who reported that the potential of the immune response can be directly linked to the blood sugar count, especially to HbA1c. It is important to mention that impaired HbA1c or blood sugar counts can be found years prior to a diabetes diagnosis. In our study, almost half of the patients in both groups showed abnormal values and had not yet been diagnosed for diabetes. This means that the actual number of diabetics in a dental practice might be even higher than expected and therefore could have atypical infection progression. Generally, for patients with known diabetes, these values are a frank sign of good or poor medicinal or dietary treatment [17].

Furthermore, it is important to differentiate such findings depending on the patients’ age, bearing in mind that the number of diabetes and comorbidities is already high in individuals older than 60. Much more interesting was the portion of diabetics in younger patients. In our study, the general group showed a diabetes prevalence of 2.1%, which is in accordance with the findings from Heidemann et al. who investigated a general German population between 18 and 79 years of age for the prevalence of diabetes mellitus. However, in our study, at 4.6%, the number of diabetics in the abscess group was even more than twice as high. Although it is not possible to tell within a retrospective study design whether these findings show that diabetes mellitus or impaired blood sugar tolerance has a direct influence on the formation of a severe odontogenic abscess, the high prevalence of diabetes among young patients further demonstrates the possibility of a diabetes-related impaired immune response which may already be relevant in younger individuals [3].

Frankly, early recognition of a dental infection helps to prevent its progress to an abscess. Many studies have focused on the mutual influence of periodontal or endodontal infections and diabetes mellitus. Diabetics show an unphysiologically increased amount of advanced glycation end products in the gingiva, which have a proinflammatory effect with an increased disposition for infections. In return, periodontal and endodontal infections can lead to systemic accumulation of inflammatory molecules which have a negative effect on insulin resistance, leading to increased HbA1c counts [6, 18–21]. Hence, it is not surprising that many study groups have shown that the progression of an infection in diabetics is much more rapid and can involve several different compartments [7, 10, 22, 23].

In conclusion, our study shows that abscesses are more likely to occur in diabetics. Most importantly, we also found that diabetics who have poor medicinal or dietary treatment proved to have the highest odds of forming a severe abscess from a dental infection. As almost half of the patients with impaired blood sugar values did not have a diabetes diagnosis, one should bear in mind that the actual number of diabetics even in general dental practice might be higher than expected. While diabetics do not show longer inpatient stays, those with abnormal blood sugar counts needed significantly longer treatment. This shows that despite the immune response which is usually impaired in diabetics due to the long-lasting early onset, it is possible to reach normal acute wound healing for these patients through sufficient medicinal or dietary treatment. Nevertheless, it should be further discussed and investigated whether patients with a known history of diabetes benefit not only from early intervention but also from early escalation of an accompanying antibiotic treatment.
